# Recurrent Aneurysmal Bone Cyst in the Metacarpal of a Child Treated With Endoscopic Curettage: A Case Report

**DOI:** 10.1155/cro/6616065

**Published:** 2026-03-16

**Authors:** Yusuke Hattori, Yohei Kawaguchi, Hiroaki Kimura, Hisaki Aiba, Hideki Okamoto, Hideki Murakami

**Affiliations:** ^1^ Department of Orthopedic Surgery, Nagoya City University Graduate School of Medical Sciences, Nagoya, Japan, nagoya-cu.ac.jp

## Abstract

**Case:**

A 5‐year‐old boy presented with right‐hand pain and was diagnosed with a bone tumor in his fifth metacarpal. Because the patient wished to resume sports activities, endoscopic curettage (ESC) was performed. The pathological diagnosis of the tumor was aneurysmal bone cyst (ABC). As recurrence was observed 1 year after the first ESC, a second ESC was performed. Postoperatively, the bone healed without complications, allowing the patient to resume physical activity.

**Conclusion:**

ESC can be performed in a tiny metacarpal of a child. The endoscopic treatment may be a feasible treatment option for ABCs in the hand.

## 1. Introduction

Aneurysmal bone cysts (ABCs) are rare, locally aggressive bone tumors characterized by cystic and osteolytic properties [1, 2]. Between 75% and 90% of cases are diagnosed within the first two decades of life, with the mean age of 13 years [3, 4]. They account for approximately 6% of all primary bone tumors [5]. Seventy percent of cases arise independently [5]. ABCs are primarily located in long bones of the lower extremity or the posterior elements of the spine [3, 6]. ABCs in the bones of the hand are particularly uncommon, accounting for only about 5% of all cases [3]. Despite various treatment options, recurrence rates within the first 2 years after treatment range from 10% to 59%, especially in young patients due to skeletal immaturity [6]. Additionally, periarticular and periphyseal lesions present a specific challenge in the management of ABCs in the hand because they are associated with difficulty in achieving complete resection, leading to a high risk of recurrence [3].

Recently, endoscopic curettage (ESC) through a small cortical window, using an arthroscope, has been described for the treatment of various benign bone tumors [5, 7, 8]. ESC is less invasive and can be an alternative to conventional curettage. Herein, we present a clinical case of ABC in the metacarpal of a child treated with ESC. This technique has not previously been described as an option for ABCs in the hand. We have conducted a literature review to provide an overview of these topics to clinicians.

## 2. Case Presentation

**Figure 1:**
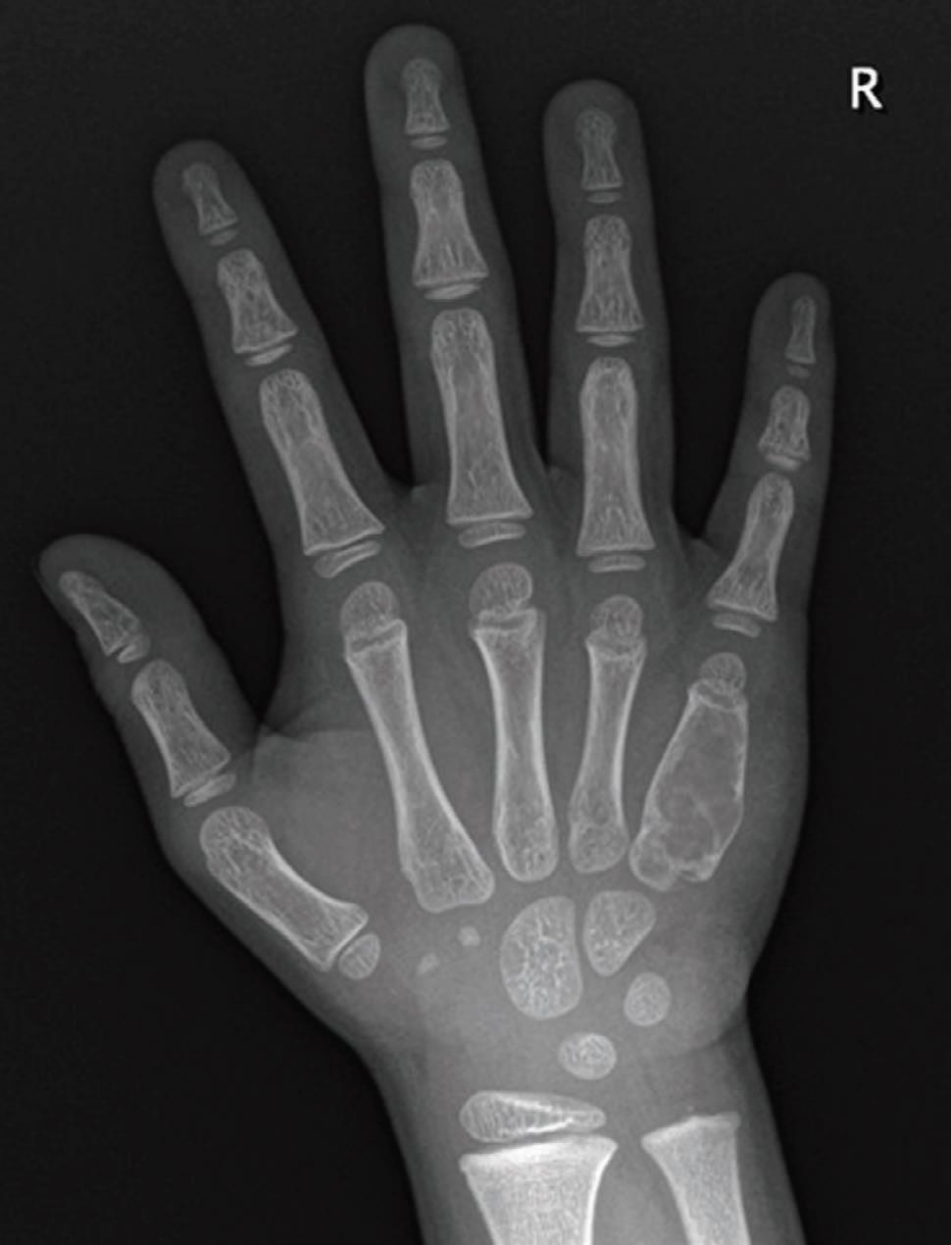
An anteroposterior plain x‐ray image shows a significant widening of the fifth metacarpal and expansion of the medullary canal. The cortex is uniformly thinned while the physis is preserved.

A 5‐year‐old boy with no comorbidities or developmental disorders presented to a local medical facility with unprovoked right‐hand pain. Before symptom onset, he actively participated in sports and had no limitations in daily activities. Radiographic examination revealed a bone tumor in the fifth metacarpal. The patient was referred to our hospital. Physical examination revealed a full range of hand motion without tenderness and swelling. Plain x‐ray images indicated significant widening of the fifth metacarpal and expansion of the medullary canal, whereas the physis remained intact (Figure 1). Magnetic resonance imaging demonstrated a multiloculated cystic lesion with multiple fluid–fluid levels, showing low‐signal intensity on T1‐weighted images and high signal intensity on T2‐weighted images (Figure 2a,b). Computed tomography demonstrated an expansile osteolytic lesion with marked cortical thinning and medullary expansion of the fifth metacarpal (Figure 2c). Although the patient was monitored as an outpatient for 1 year, the tumor continued to grow. Based on these characteristic radiological findings, a benign bone tumor such as a simple bone cyst, enchondroma, and ABC was strongly suspected. Because curettage allows both treatment and histological diagnosis, we planned a single‐stage therapeutic ESC without preoperative biopsy or intraoperative frozen section. After discussion of the treatment options, the patient′s parents provided written informed consent for surgery.

Figure 2Magnetic resonance images demonstrate a multiloculated cystic lesion with multiple fluid–fluid levels: (a) T1‐weighted image shows predominantly low signal intensity within the cystic cavities with fluid–fluid levels. (b) T2‐weighted image shows hyperintense cystic cavities with multiple fluid–fluid levels. Internal septa and the cyst wall are depicted as low–signal‐intensity structures outlining the lesion. (c) Coronal computed tomography image shows an expansile osteolytic lesion of the fifth metacarpal with marked cortical thinning and medullary expansion.

**Figure:**
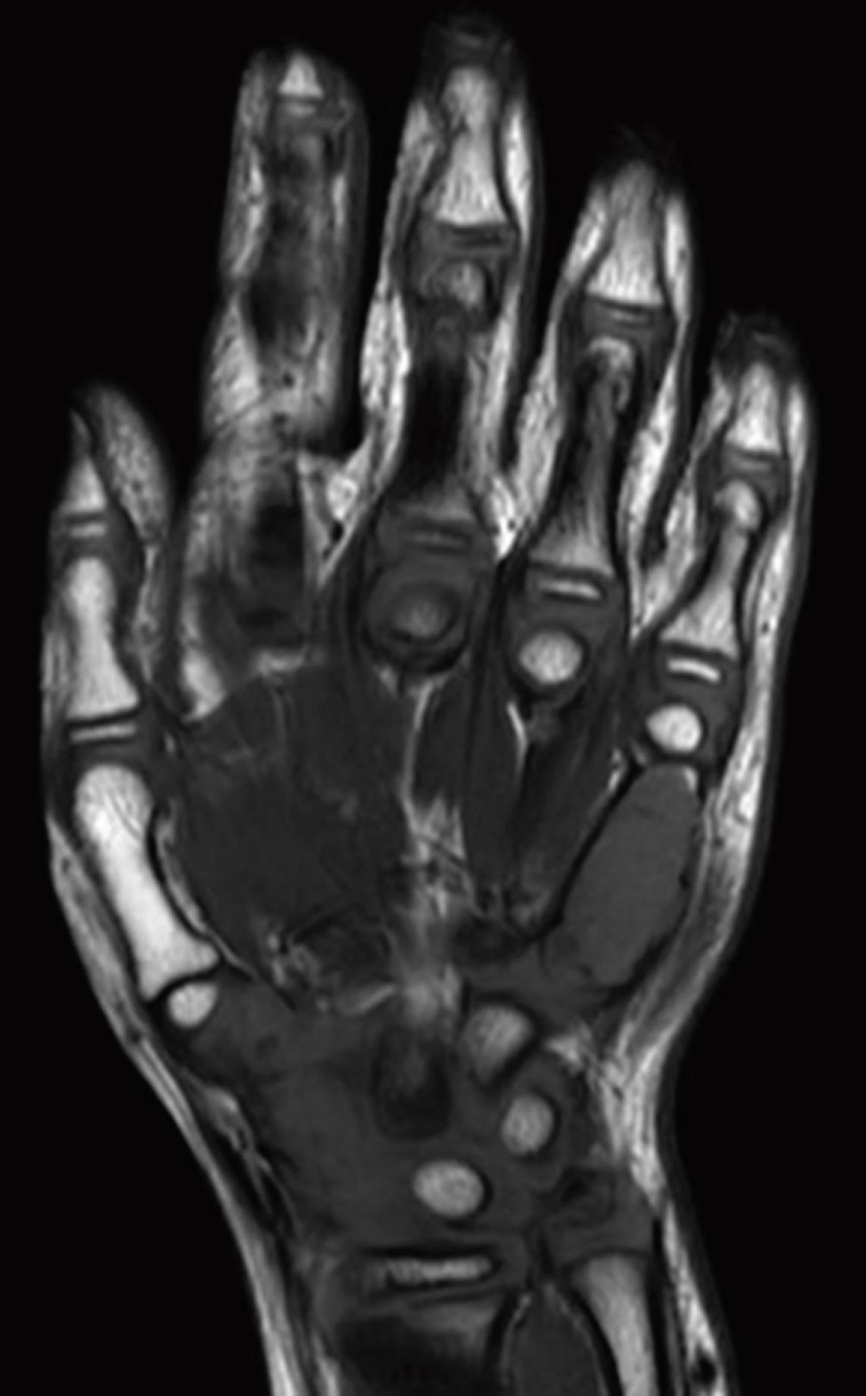
(a)

**Figure:**
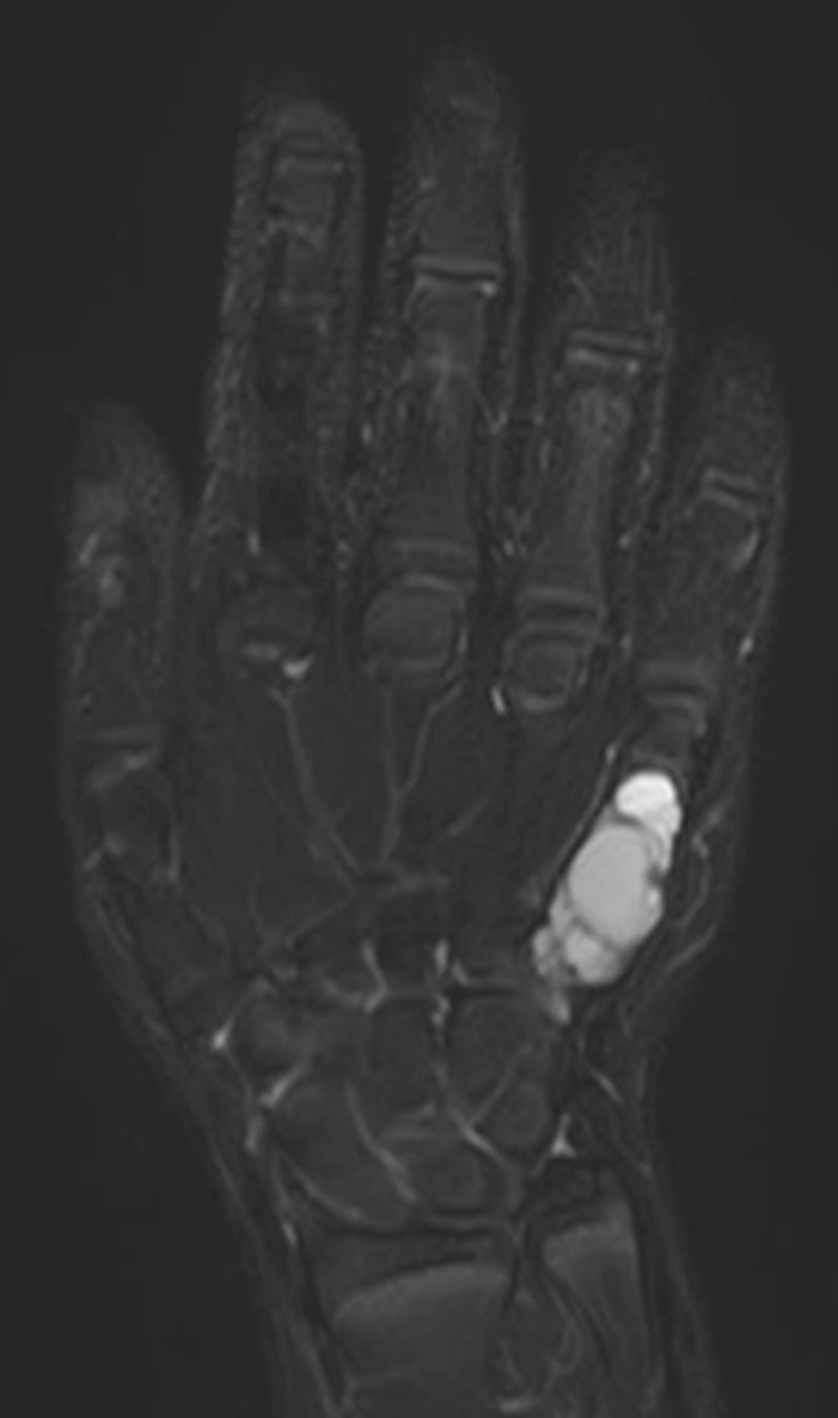
(b)

**Figure:**
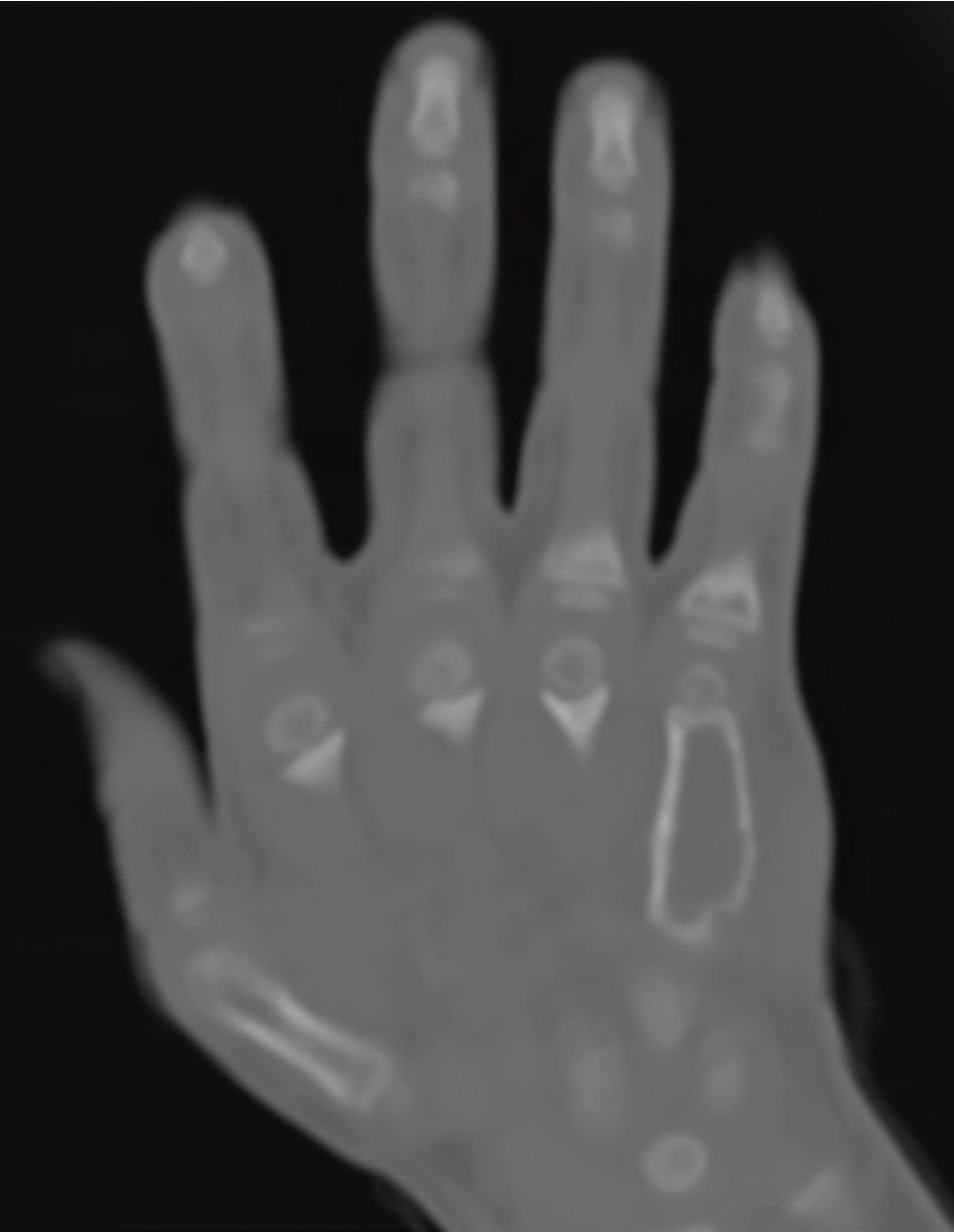
(c)

One year after the initial visit, the patient underwent ESC under general anesthesia with tourniquet control. On the plain x‐ray image, the longitudinal diameter of the tumor was about 22 mm. Two portals were created on the ulnar side and one on the radial side through 5‐mm‐long incisions with blunt dissection (Figure 3a). The cortical bone was then penetrated using a 1.8‐mm‐diameter K‐wire, and a 1.5‐mm straight arthroscope was inserted (Figure 3b). The tumor tissue was curetted using a small sharp curette and an electric shaver. The insertion holes for the arthroscope and other instruments were interchanged until normal bone was observed in the medullary cavity (Figure 4). No bone grafts or adjuvant therapies were administered. Pathological diagnosis confirmed the tumor as an ABC (Figure 5).

Figure 3Appearance of endoscopic curettage: (a) Two portals are created on the ulnar side and one on the radial side; (b) Surgical maneuver with an arthroscope and other instruments.

**Figure:**
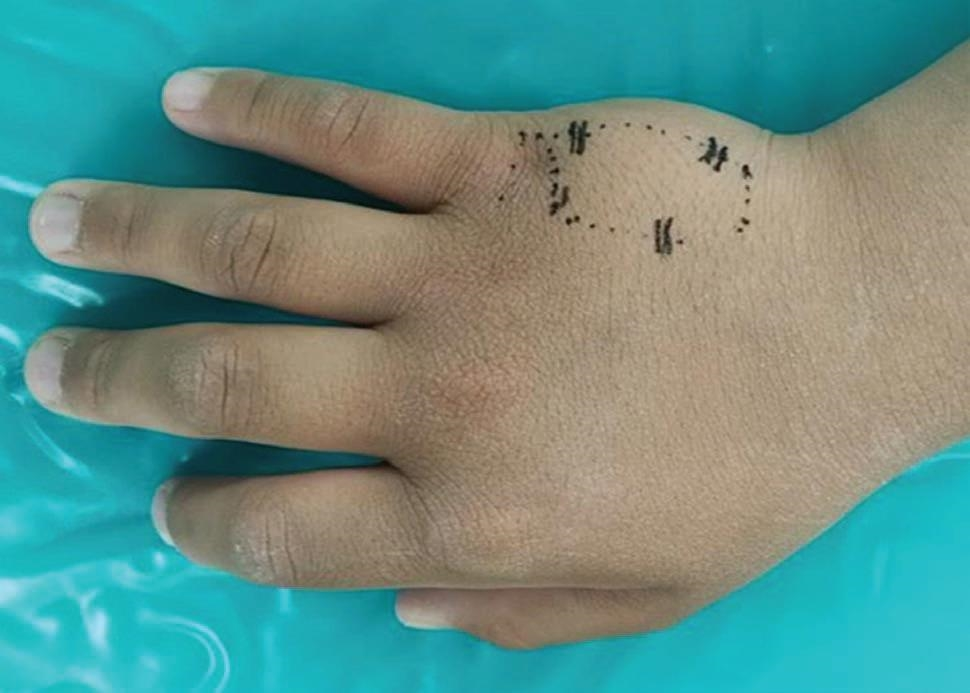
(a)

**Figure:**
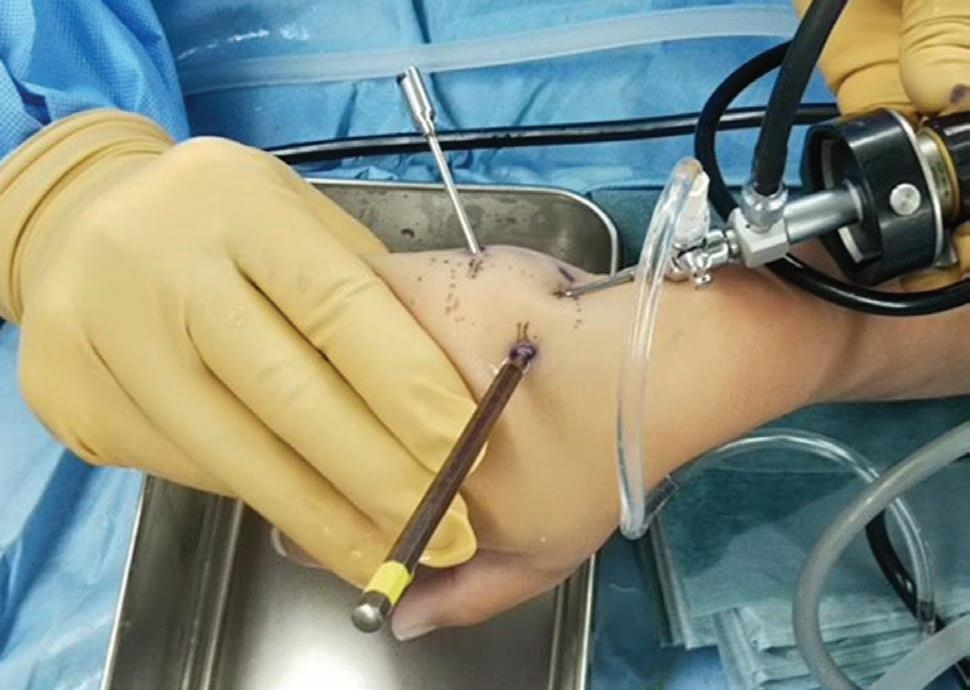
(b)

Figure 4Endoscopic images: (a) The medullary cavity is covered with tumor tissue. The dotted area shows tumor tissue curetted using a small sharp curette; (b) Curettage is extended until normal bone is visible in the medullary cavity.

**Figure:**
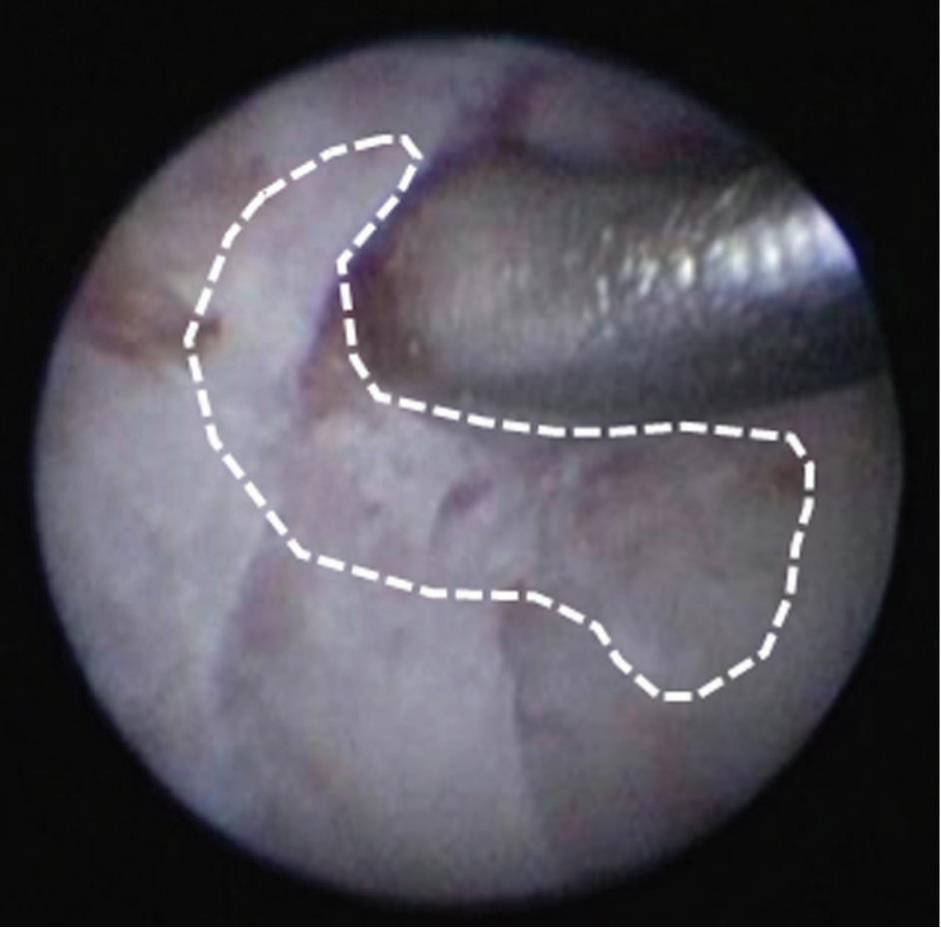
(a)

**Figure:**
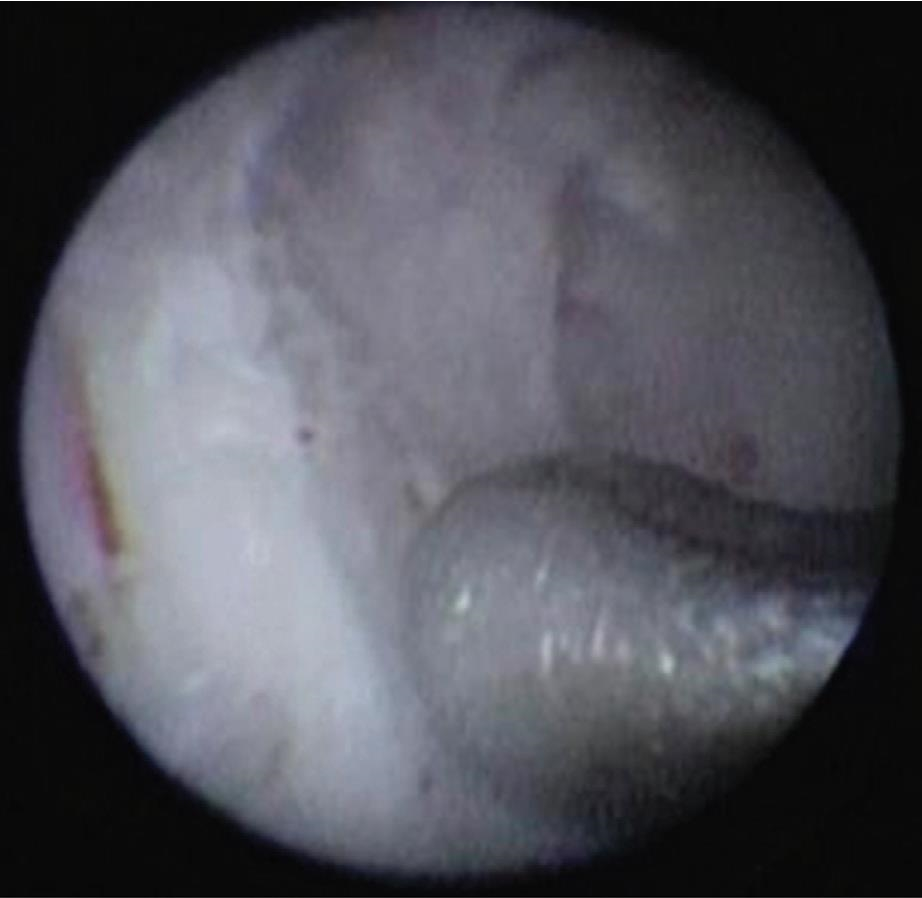
(b)

Figure 5Gross and histological appearance of tumor tissue: (a) Curetted tumor tissue; (b, c) Cystic wall containing numerous giant cells (arrows) (hematoxylin–eosin, (b) Low‐power field, and (c) High‐power field).

**Figure:**
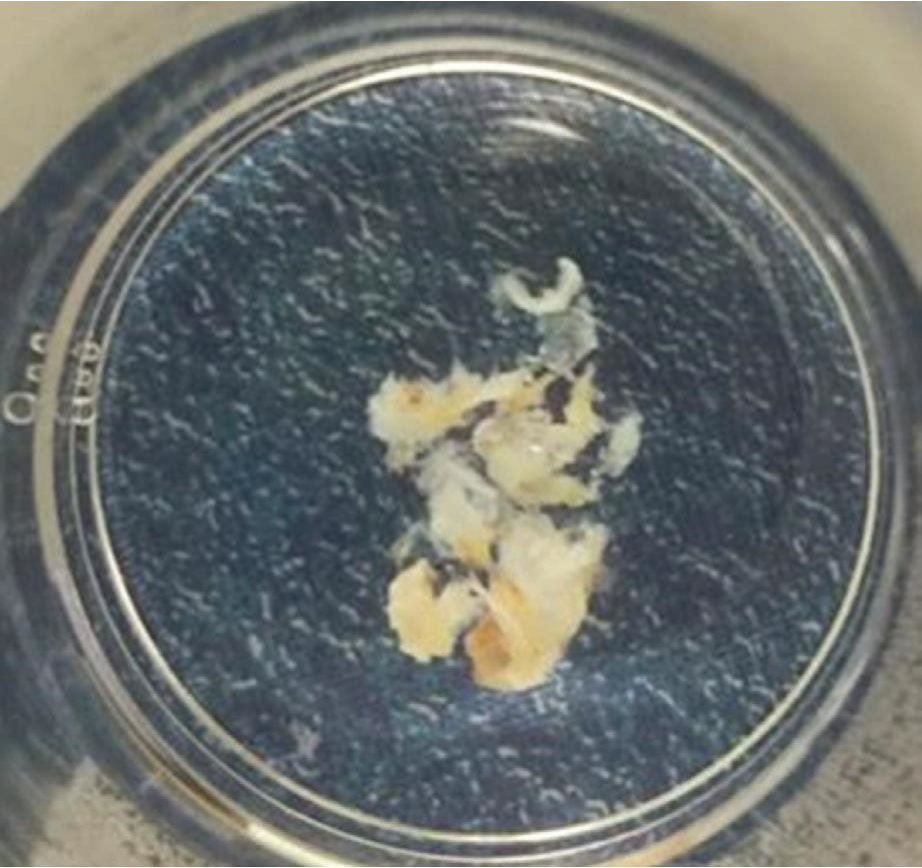
(a)

**Figure:**
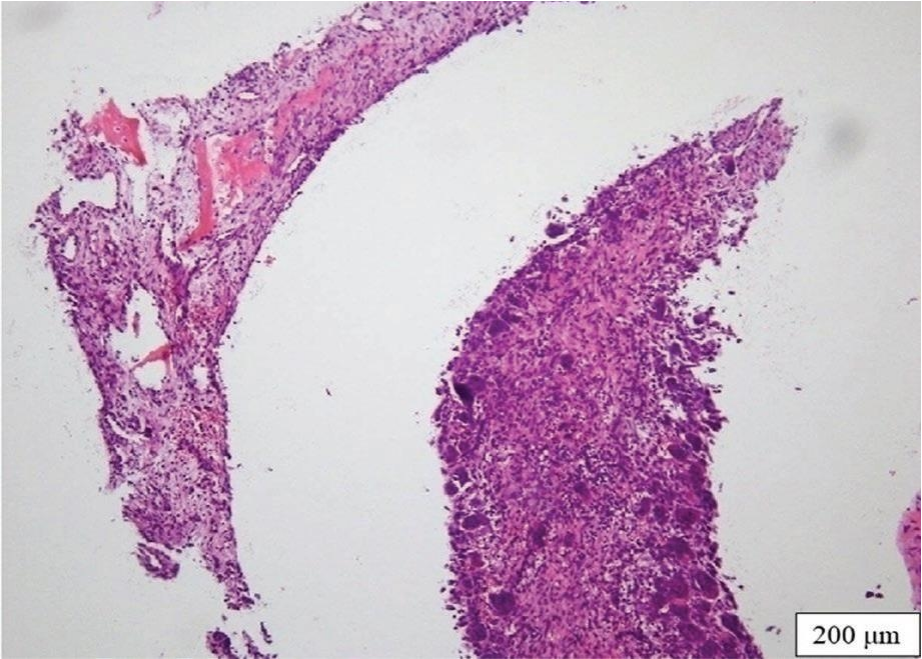
(b)

**Figure:**
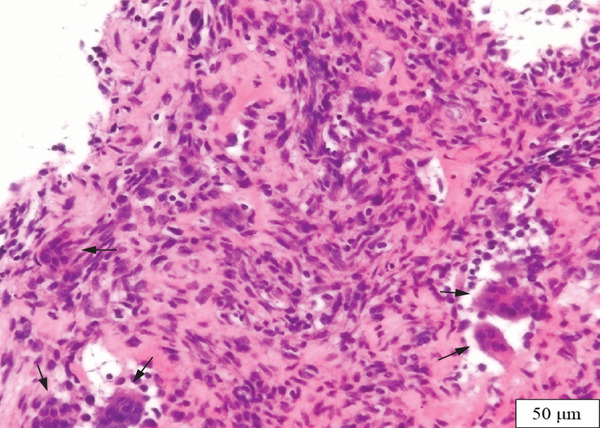
(c)

**Figure 6:**
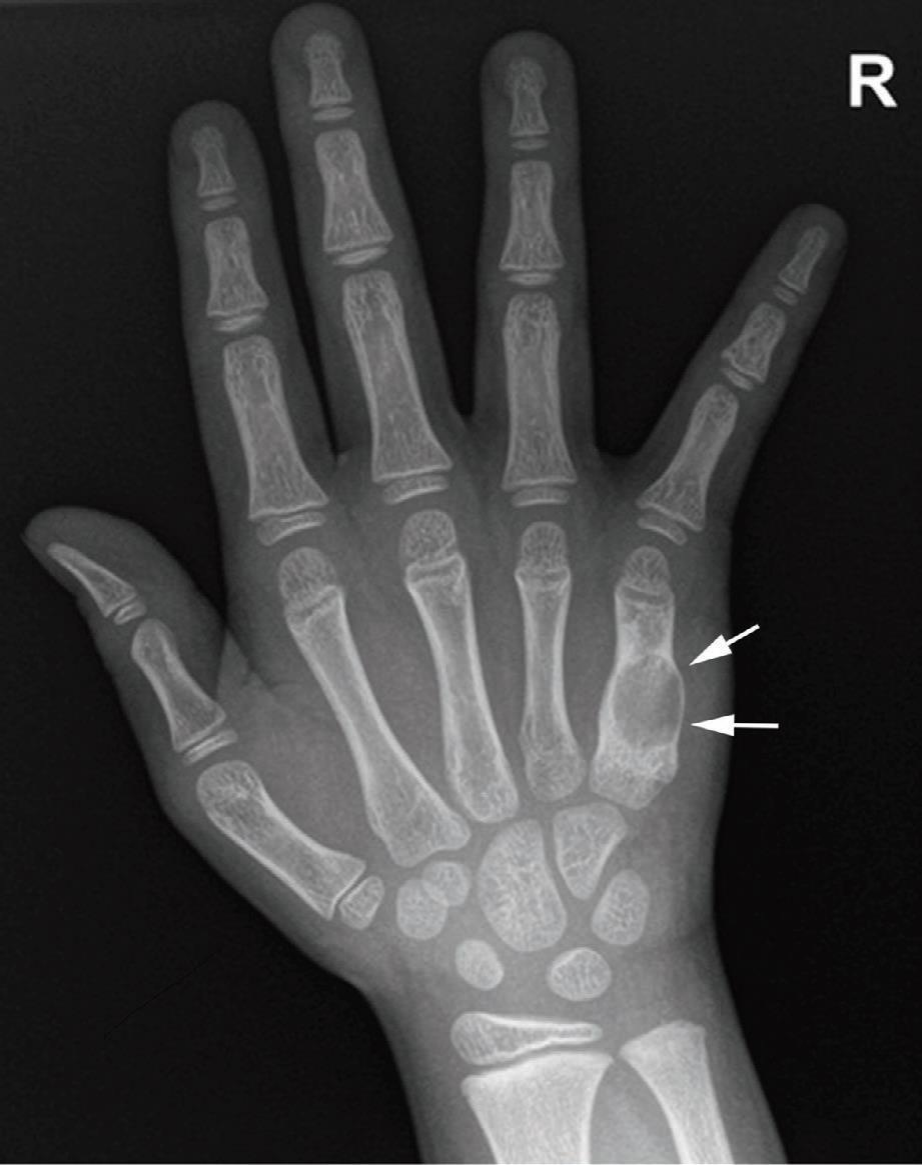
One year after the first endoscopic curettage, an anteroposterior plain x‐ray image shows renewed osteolytic changes with medullary expansion and cortical thinning (arrows), consistent with tumor recurrence in the fifth metacarpal.

**Figure 7:**
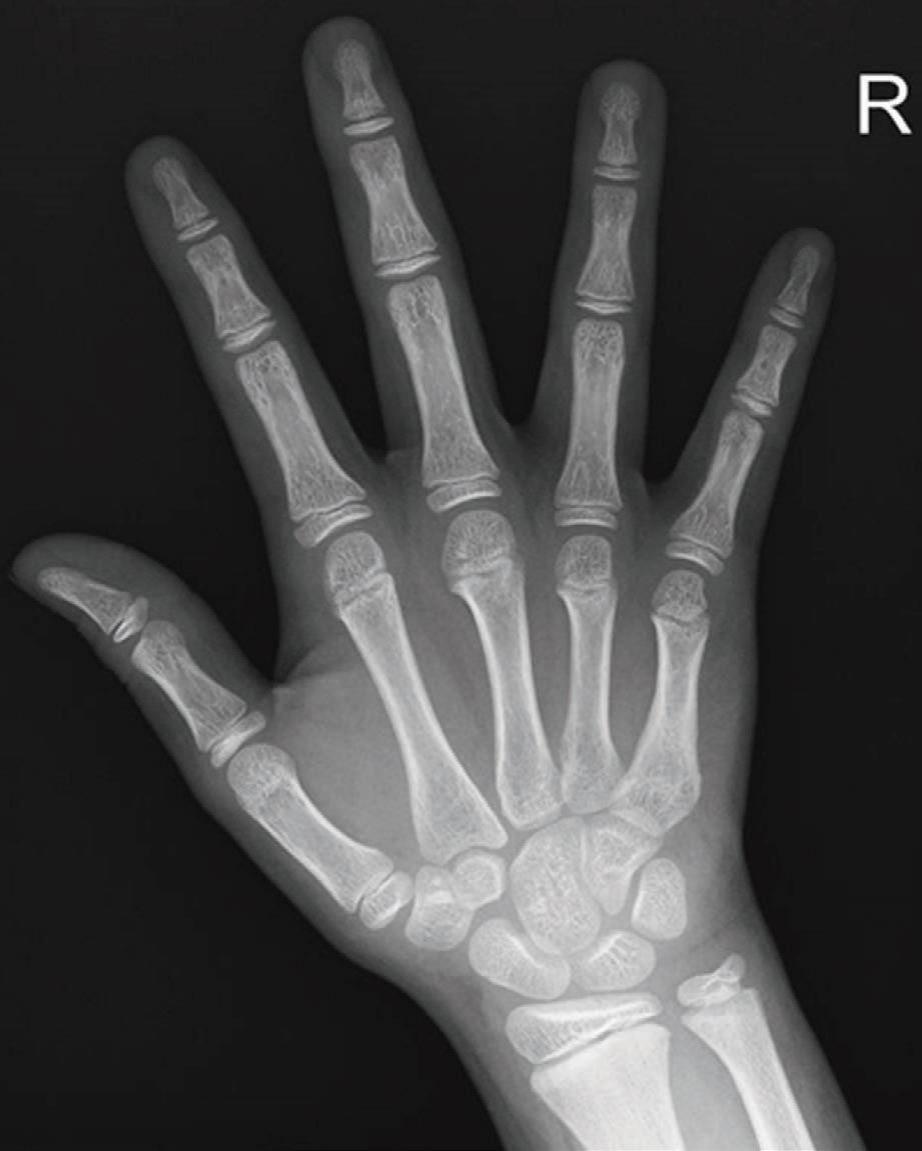
Three years after the second endoscopic curettage, an anteroposterior plain x‐ray image demonstrates reossification of the medullary cavity, cortical thickening, restoration of normal bone contours, and the absence of progressive osteolytic changes, confirming bone healing without recurrence.

Postoperatively, the bone tended to consolidate and heal. The patient was asymptomatic and successfully returned to sports activities. However, tumor recurrence was suspected on plain x‐ray images 1 year postoperatively (Figure 6). Magnetic resonance imaging was subsequently performed and confirmed recurrent cystic changes consistent with ABC (Figure S1a,b), despite the absence of clinical symptoms. A second ESC was performed using one portal on both the ulnar and radial sides. The same procedure as that used for the initial surgery was performed to scrape the tumor. No bone grafts or adjuvant therapy were administered. Pathological diagnosis confirmed the tumor as a recurrence of ABC. Postoperatively, radiographic evidence of bone consolidation was used as the primary criterion for permitting return to sports activities. The patient successfully resumed sports without pain or functional impairment. No adverse events, including infection, neurovascular injury, or postoperative stiffness, were observed during follow‐up. At 3 years after the second surgery, the patient played baseball without pain, and no recurrence was observed on plain x‐ray images (Figure 7). The patient′s parents reported satisfaction with the treatment outcome.

## 3. Discussion

The treatment options for ABCs vary widely and include surgical removal of the entire lesion or curettage of the lesion, with or without bone grafting. Other treatment options include cryosurgery, sclerotherapy, and adjuvant therapies, such as peroxide and phenol [3, 6, 9]. ABCs in the hand have a lower recurrence rate than those in other locations [9]. Therefore, less aggressive treatment should be considered.

ESC has been used for treating various bone tumors, including ABCs [5, 7, 8]. This procedure can be performed through small incisions, making it esthetically superior and less invasive than conventional open curettage [5]. This endoscopic approach also has the advantage of accurately assessing tumor resection by directly visualizing the bone marrow cavity [5]. Smolle et al. reported that regardless of the surgeon′s experience level, ESC could be superior to conventional curettage for complete tumor removal [10]. Despite the prolonged operation time required for hole preparation and careful, piece‐by‐piece curettage to avoid excessive curettage or leaving residual tumors [5, 10], ESC may be a good alternative for treating benign bone tumors.

Although some surgeons have performed ESC with bone graft for treating benign bone tumors [7, 11], our experience has shown that this technique can lead to new bone formation without the need for bone grafts [5, 8]. Autologous bone grafting is associated with limitations, such as limited bone supply and donor site morbidity [12]. Bone prostheses have disadvantages related to their absorption rates and costs [13]. However, ESC can directly stimulate the bone marrow cavity and facilitate new bone formation [5]. This may be an additional advantage of this technique.

In this case, the patient′s age of under 10 years and tumor contact with the physis were present. These factors increase the risk of recurrence due to open growth plates and concern for physeal damage [5]. However, we opted for ESC because of its advantage of direct visualization of the bone marrow cavity, allowing for precise tumor resection [5, 10]. Furthermore, previous studies reported that more aggressive treatment, such as burring, peroxide, and tumor resection with autologous bone graft, was performed for recurrent cases [3, 6]. These options have concerns about limited range of motion and donor site morbidity [6]. Conversely, ESC is a minimally invasive procedure that preserves the surrounding soft tissue and achieves good functional recovery [5]. Another minimally invasive treatment option for ABCs is radio‐opaque gelified ethanol sclerotherapy [14]. Although this percutaneous technique has shown promising results, it typically requires multiple treatment sessions and is associated with repeated radiation exposure. In contrast, ESC allows direct visualization of the lesion and can be completed in a single procedure. Considering these advantages, we selected ESC as the treatment option for this case. The 5‐mm skin incisions were minimally visible, leading to high patient satisfaction.

Despite the minimally invasive advantages of ESC, this case demonstrated local recurrence after the first procedure. This patient had high‐risk factors, including a very young age and close proximity of the lesion to the physis. In addition, the small size of the metacarpal bone in very young children results in a limited working space, which may further increase the risk of incomplete tumor removal. Therefore, given the recurrence observed after the initial ESC, vigilant postoperative monitoring with radiographic imaging is crucial for patients at high risk of recurrence. Finally, because this report describes a single case, the level of evidence is inherently limited.

In conclusion, we encountered a case of ABC in a tiny metacarpal of a 5‐year‐old treated with ESC. Despite recurrence requiring a second procedure, ESC may be a feasible and minimally invasive option for ABCs in the hand.

## Author Contributions

Yusuke Hattori: writing – original draft and data curation. Yohei Kawaguchi: methodology, investigation, writing – review and editing. Hiroaki Kimura: writing – review. Hisaki Aiba: investigation and visualization. Hideki Okamoto: investigation and visualization. Hideki Murakami: writing – review and editing and conceptualization.

## Funding

This study supported by a grant‐in‐aid for scientific research (22K09405) from the Ministry of Education, Culture, Sports, Science, and Technology of Japan.

## Disclosure

All authors have read and approved the final version of the paper.

## Ethics Statement

Ethics committee approval was not required because of the nature of this case report. Patient anonymity was maintained according to the Declaration of Helsinki.

## Consent

Written informed consent for the publication of this case was obtained from the patient′s parents.

## Conflicts of Interest

The authors declare no conflicts of interest.

## Supporting information


**Supporting Information** Additional supporting information can be found online in the Supporting Information section. Figure S1: Magnetic resonance images obtained 1 year after the first endoscopic curettage confirm local recurrence: (S1a) T1‐weighted image shows a cystic lesion with predominantly low signal intensity and (S1b) T2‐weighted image shows hyperintense cystic cavities with multiple fluid–fluid levels, consistent with recurrent aneurysmal bone cyst.

## Data Availability

The data that support the findings of this study are available from the corresponding author upon reasonable request.
