# Supplementary figures and images for: Recurrent Aneurysmal Bone Cyst in the Metacarpal of a Child Treated With Endoscopic Curettage: A Case Report
Source: Case Rep Orthop. 2026 Mar 16;2026:6616065. doi: 10.1155/cro/6616065 (PMC13409334; doi:10.1155/cro/6616065)

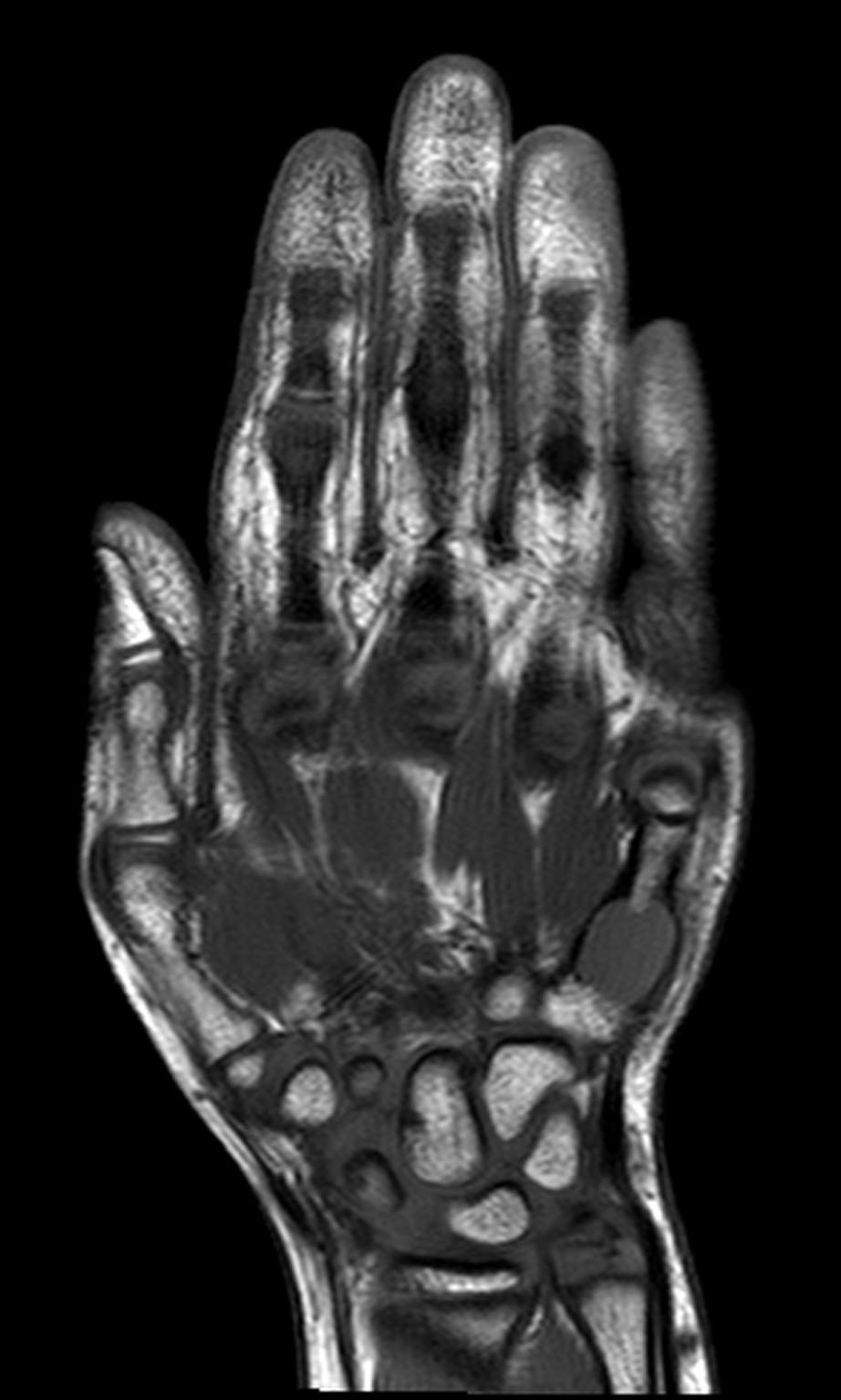

Supplement: Supplementary file 1 — Supporting Information Additional supporting information can be found online in the Supporting Information section. Figure S1: Magnetic resonance images obtained 1 year after the first endoscopic curettage confirm local recurrence: (S1a) T1‐weighted image shows a cystic lesion with predominantly low signal intensity and (S1b) T2‐weighted image shows hyperintense cystic cavities with multiple fluid–fluid levels, consistent with recurrent aneurysmal bone cyst. [file CRO-2026-6616065-s001.zip › 6616065.f1/Supplementary Figure S1a.tif]

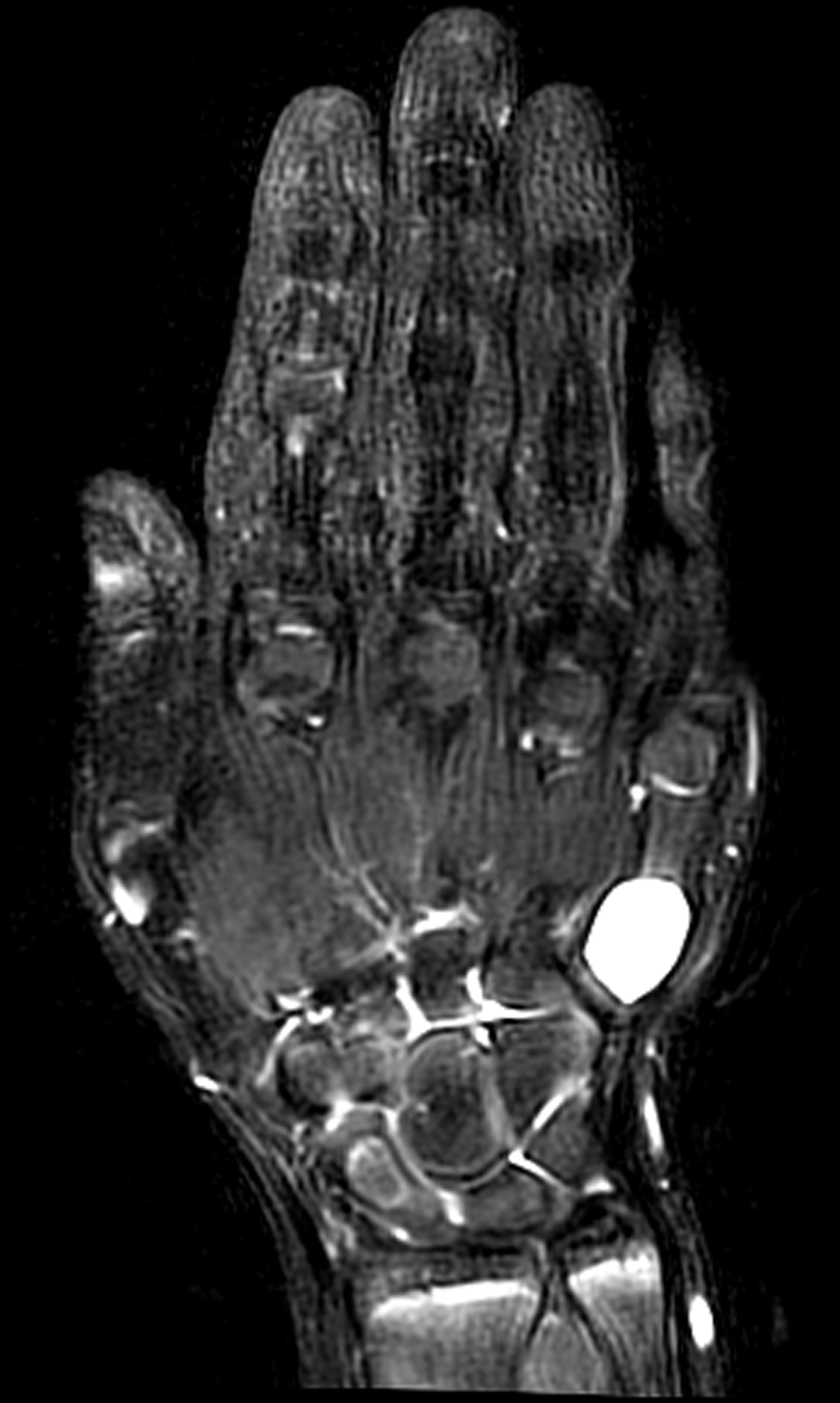

Supplement: Supplementary file 1 — Supporting Information Additional supporting information can be found online in the Supporting Information section. Figure S1: Magnetic resonance images obtained 1 year after the first endoscopic curettage confirm local recurrence: (S1a) T1‐weighted image shows a cystic lesion with predominantly low signal intensity and (S1b) T2‐weighted image shows hyperintense cystic cavities with multiple fluid–fluid levels, consistent with recurrent aneurysmal bone cyst. [file CRO-2026-6616065-s001.zip › 6616065.f1/Supplementary Figure S1b.tif]
